# Microwave ablation eustachian tuboplasty: a preliminary investigation with long-term follow-up

**DOI:** 10.1186/s40463-021-00520-2

**Published:** 2021-06-24

**Authors:** Zhengcai Lou, Zihan Lou, Junzhi Sun, Zhengnong Chen, Shankai Yin

**Affiliations:** 1 Department of Otorhinolaryngology, Yiwu Central Hospital, 699 Jiangdong Road, Yiwu City, 322000 Zhejiang Provice China; 2 Department of Otolaryngology-Head and Neck Surgery, Shanghai Jiao Tong University Affiliated Sixth People’s Hospital, Otolaryngology Institute of Shanghai Jiao Tong University, 600 Yishan Road, Shanghai, 200233 China; 3 Otolaryngology Institute of Shanghai Jiao Tong University, Shanghai, 200233 China

**Keywords:** Eustachian tube dysfunction, Microwave ablation, Tympanometry, Tympanic membrane, Valsalva maneuver

## Abstract

**Objectives:**

This study was performed to evaluate the efficacy of microwave ablation (MWA) eustachian tuboplasty for the treatment of patients with retracted tympanic membrane (TM) due to eustachian tube dysfunction (ETD).

**Methods:**

This was a prospective study of 20 patients with ETD (middle ear atelectasis) who underwent MWA eustachian tuboplasty. Outcomes included the ability to perform a Valsalva maneuver, audiometry results, tympanometry results, ETD Questionnaire (ETDQ-7) score, and TM status.

**Results:**

Eighteen patients (18 ears) were included in this study. There were statistically and clinically significant improvements in the mean ETDQ-7 score at 6 months postoperatively (change in mean score of 16.7 ± 3.6, *P* < 0.001) and at 30 months postoperatively (change in mean score of 18.9 ± 2.9, *P* < 0.001).

Type A tympanogram was obtained in 27.8% of patients (5/18) at 6 months postoperatively, and in 77.7% at 30 months postoperatively. A Valsalva maneuver was possible in 72.2% of patients at 6 months postoperatively and in 88.9% of patients at 30 months postoperatively. In addition, the ears of 13 patients (72.2%) showed both normal tympanograms and TM at 30 months postoperatively. Interestingly, 38.5% of patients (5/13) exhibited complete sclerosis of the pars tensa. None of the patients experienced severe MWA-related complications during follow-up.

**Conclusions:**

MWA eustachian tuboplasty is a feasible alternative to conventional tuboplasty, and can improve subjective and objective outcomes in patients with ETD for up to 30 months following treatment. In addition, this study showed that the extent of sclerotic plaque increased over time, whereas the extents of atrophy and tensa retraction decreased following tuboplasty in most patients.

**Graphical abstract:**

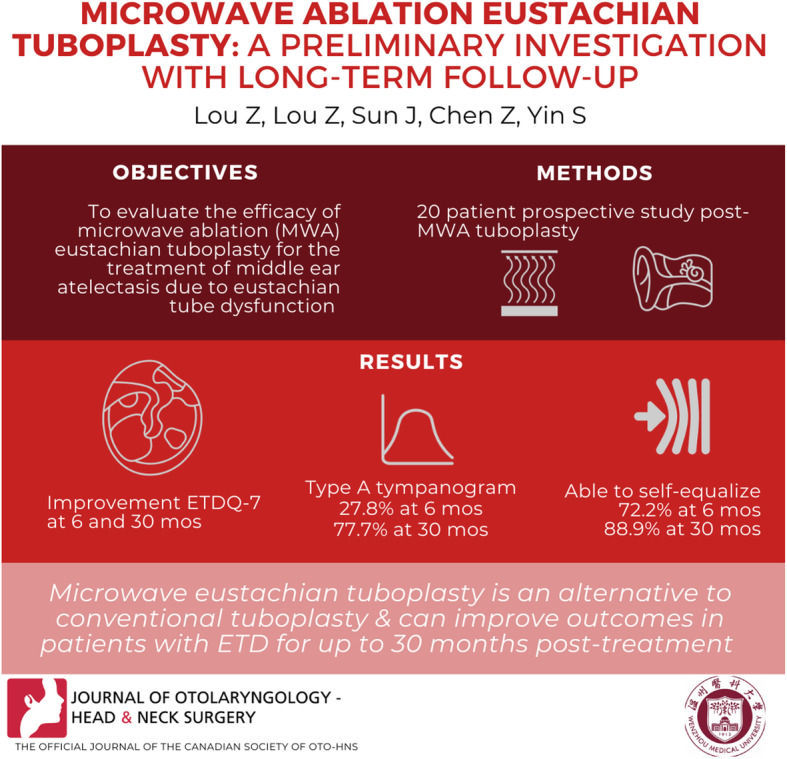

## Introduction

Eustachian tube dysfunction (ETD) is a frequent problem in otology clinics that can lead to various otological manifestations and complications, such as serous otitis media, tympanic membrane (TM) retraction, adhesive otitis, and atelectasis of the middle ear [1–3]. ETD refractory to medical therapy has been treated primarily by bypassing the eustachian tube (ET) and ventilating the middle ear directly through the TM with a ventilation tube and then restoring the TM to its normal contours. However, ventilation tubes (VTs) are subject to problems with crusting, infection, obstruction, and unplanned extrusion [2]. Recent studies have identified the cartilaginous portion of the ET as the most frequent site of dysfunction [1–6]. In addition, the familiarity with nasal endoscopy among otolaryngologists has led to the development of new options for the treatment of ETD, including laser eustachian tuboplasty [2–4] and microdebrider-assisted tuboplasty [5]. Notably, balloon dilation of the cartilaginous ET has produced encouraging results [5–9]. Although balloon eustachian tuboplasty is becoming increasingly popular and has demonstrated high efficacy in Europe and the USA [10], balloon tuboplasty has not been widely adopted because of its high cost and limitations involving medical insurance in China.

We have performed eustachian tuboplasty using microwave ablation (MWA) to treat chronic ETD since 2013. MWA is a unique application of dielectric heating, in which the tissue is the dielectric material. Dielectric heating occurs when an alternating electromagnetic field is applied to an imperfect dielectric material [11]. MWA heating is governed by the oscillation of polar molecules in the induced electromagnetic field [12], which results in more rapid tissue heating around the microwave antenna. This allows shorter treatment times and a larger zone of ablation [13]. MWA has been used to treat epistaxis and remove nasal benign lesions with minimal side effects in otolaryngology practice in recent years [14–17]. The present study was performed to examine the efficacy of MWA of the cartilaginous lumen for the treatment of patients with chronic ETD.

## Methods

### Patient enrollment and assessment

**Fig. 1:**
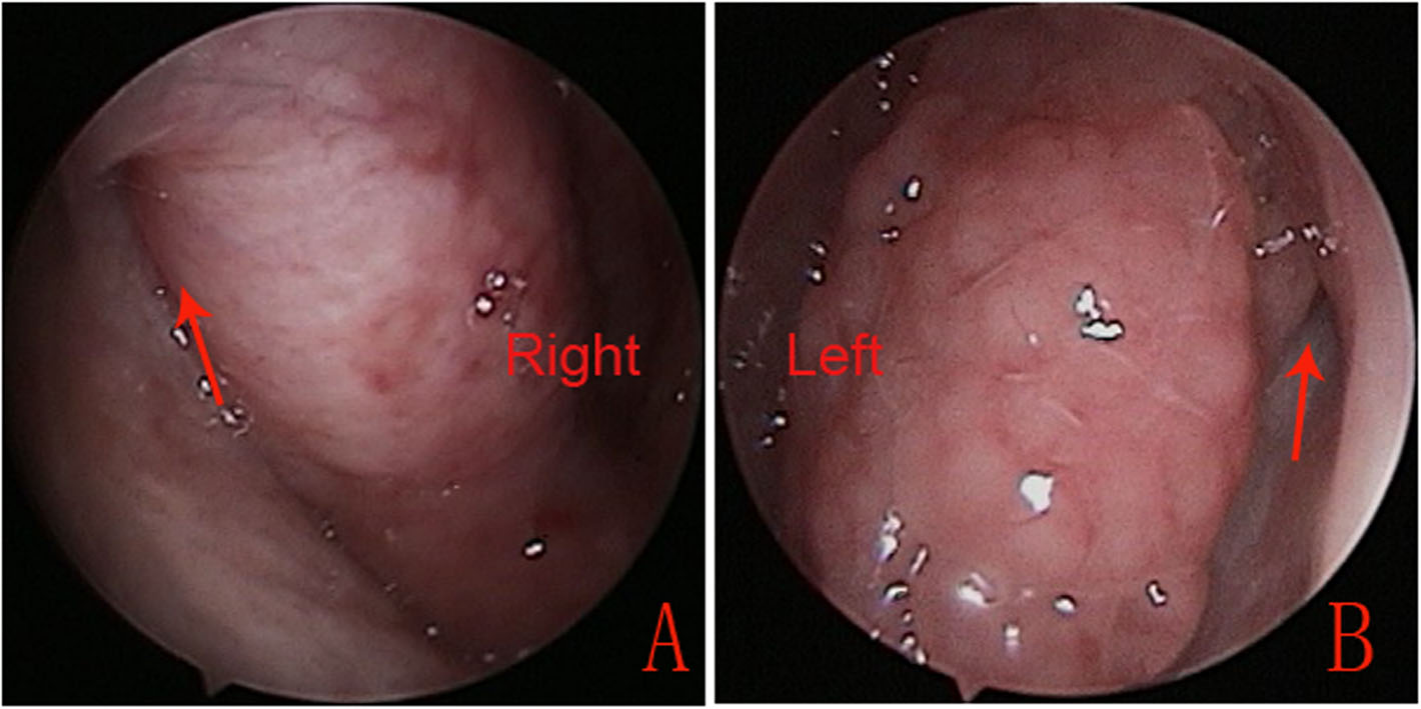
Endoscopic examination showed hyperplastic mucosa and a narrow tubal lumen (red arrows) in the cartilaginous portion of the ET (**a** and **b**)

Adult patients with evidence of chronic ETD were enrolled in this prospective study. The inclusion criteria were as follows: age ≥ 18 years; unilateral chronic dilatory ETD with persistent TM retraction (Sade Grade II or III) and significant hypertrophic mucosa in the ET orifice (Fig. 1); ≥ 3 ETD symptoms (ear pain, ear pressure, tinnitus, cracking or popping in ears, muffled hearing, feeling that the ears are clogged) [9], and duration of symptoms > 12 months; history of failed conservative medical treatment; no existing TM perforation or VT at the time of surgery (regardless of previous VT history); presence of type B or C tympanogram and negative Valsalva maneuver result; preference for non-conservative treatment and willingness to undergo follow-up for ≥30 months; and ETD Questionnaire (ETDQ-7) score ≥ 2.1. Exclusion criteria were as follows: the presence of a TM perforation or tympanostomy, prior surgical interventions to dilate the ET; anatomical conditions that might prevent transnasal access to the ET; planned concomitant nasal or ear procedures; history of radiation therapy or major head/neck surgery within the preceding 4 months; fluctuating sensorineural hearing loss; active chronic or acute otitis media; and allergies or reflux disease not controlled with medication. In addition, all participants were required to undergo computed tomography analysis of temporal bones. Participants with evidence of carotid artery dehiscence and nasopharyngeal carcinoma were excluded.

Failed medical therapy was defined as a minimum of either 4 weeks of daily intranasal steroid spray and no improvement during a 3-month follow-up or ≥ 1 completed course of an oral steroid within 12 months before enrollment in the study. The Valsalva maneuver was defined as a self-induced effort to insufflate air into the middle ear through the ET. The Valsalva maneuver result was objectively recorded as positive if the investigator could see TM movement or hear the sound of air flow within the TM. The ETDQ-7 is a validated, standardized, seven-item patient-reported questionnaire for assessing symptom severity associated with ETD [18]. The questionnaire is not specific for laterality of the affected ear(s). Each item is assessed on a scale of 1 (no problem) to 7 (severe problem). An overall score, which is the mean of the seven item scores, is then calculated. The questionnaire regards ETD as individual symptom scores of > 2.1 and a total score of > 14.5. Scores in the range of 1–2 indicate no to mild symptoms, 3–5 indicate moderate symptoms, and 6–7 indicate severe symptoms.

The pure-tone average was calculated as the mean of behavioral thresholds at 500, 1000, 2000, and 3000 Hz. The air-bone gap (ABG) was determined as the mean of the differences between air-conduction thresholds and bone-conduction thresholds at 500, 1000, 2000, and 3000 Hz and was measured preoperatively and at 6 and 30 months postoperatively.

### Surgical technique

MWA was performed using the EBH-IV device (Shanghai Xiyu Electromechanical System Co. Ltd., China). The microwave device consisted of a 2450-MHz generator, an output cable, multiple single cooled-shaft microwave antenna attachments, and a footplate-operated switch. The microwave antenna tip was designed contact-type in this study. The output power from the generator of the EBH-IV device ranges from 0 to 100 W. In this study, an output power of 60 W was used. A footplate-operated switch was used to control the ablation time (maximum of 20 s), as well as the length, width, and depth of penetration of the thermal lesion. Ablation was immediately terminated if the switch was deactivated. In addition, repeated MWA cycles of 2–3 s were implemented to reduce the severity of the thermal lesion and the risk of side effects [13–16]. Ablation was terminated when the ablated zone achieved a gray-white color. The microwave antenna was 9 cm in length and had an outer diameter of 3 mm. The antenna tip was a split-type double needle with an upward curve. The microwave antenna tip used to contact the hyperplastic mucosa is available with a diameter of 1 mm and length of 2 mm [15, 16].

Patients were placed in the supine position and administered general anesthesia with endotracheal intubation. Cotton pledgets were soaked in 4% (w/v) lidocaine and adrenaline, and then placed in the nasal cavity three times at 5-min intervals to reduce bleeding and improve visualization. A 30° endoscope was used to evaluate the appearance of the ET.

**Fig. 2:**
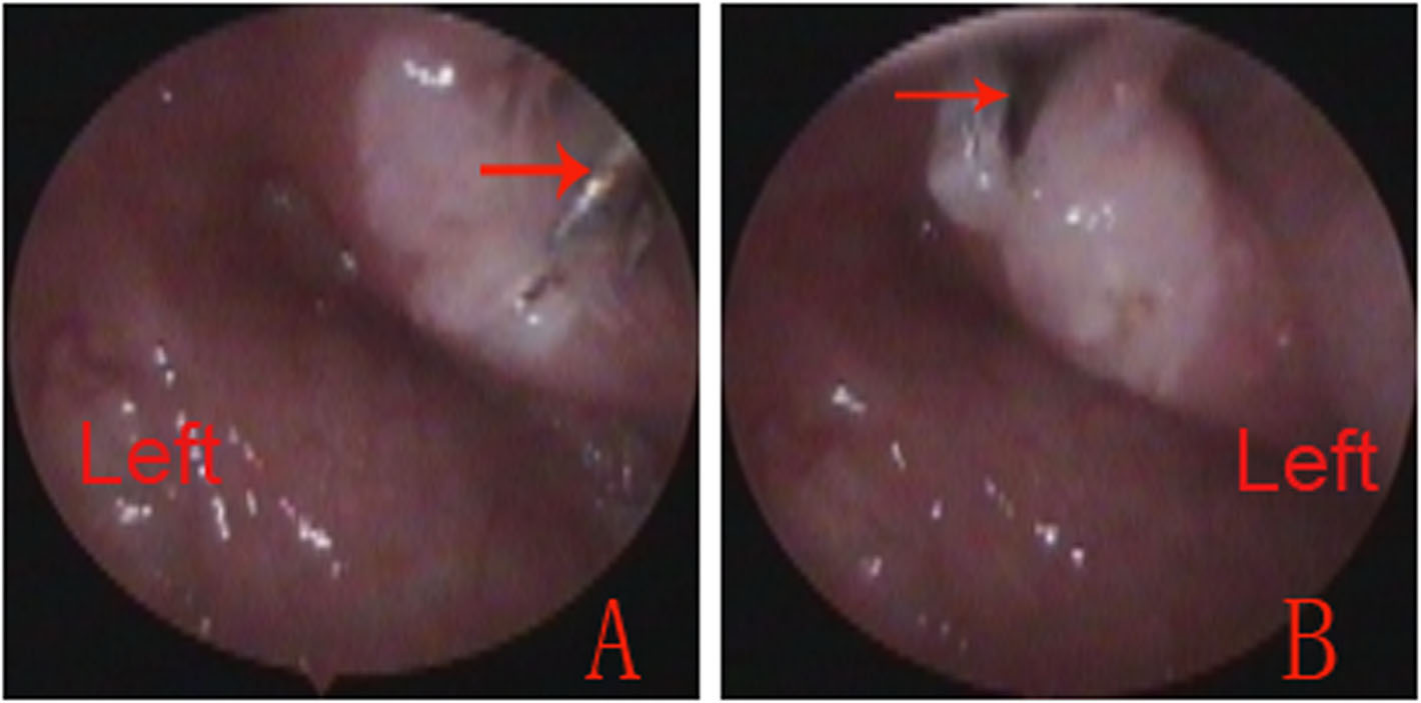
Microwave ablation eustachian tuboplasty (**a** and **b**). Red arrows indicate the microwave antenna tip

Patients underwent MWA tuboplasty targeting the hypertrophic tissue of the ET orifice. Ablation of hypertrophic mucosa and submucosa was begun over the leading edge of the medial cartilaginous lamina at the free border of the posterior cushion of the ET. The ET cartilage could be readily palpated with a straight suction or microwave antenna tip. We performed multiple and pointed tissue ablations on the posterior cushion. The ablation zone extended from the orifice of the ET, 3 mm distally into the posterior wall of cartilaginous tubal lumen in cases of severe hyperplastic mucosa. The number of ablation applications depended on the size of the treated area until the ablated zone had a gray-white appearance (Fig. 2). The extent of overall tissue ablation was determined by the bulk of hyperplastic mucosa.

Notably, all tissue removal remained superficial to this cartilage, which served as the deep margin of surgical dissection throughout the procedure. Cartilage ablation may have led to thinning and weakening of cartilage elasticity in a few patients with severe obstructive disease. However, injury to the mucosa along the anterior tubal wall was avoided to prevent postoperative scarring and stenosis of the ET orifice. No nasal or cartilaginous tube packing was used following ablation. Patients were discharged within 24 h of surgery with oral antibiotics and instructions to perform daily nasal saline irrigations until the 1-week postoperative visit.

### Follow-up and outcome measures

Once-weekly postoperative visits were scheduled in the outpatient clinic during each of the first 2 weeks. Follow-up visits were then scheduled at 1, 6, 12, 24, and 30 months postoperatively. These visits included assessments of the TM, ET, tympanometry results, and ETDQ-7 score. At each visit, TM morphology and motility were recorded with a 0° endoscope in a blinded manner by a surgeon who was not involved in the treatment. The nasal cavity, ET orifice, and nasopharynx were evaluated by videoendoscopy.

Intranasal corticosteroid sprays were continued for 6 weeks to reduce postoperative mucosal edema. Three patients with suspected laryngopharyngeal reflux were advised to continue 20 mg omeprazole daily for at least 8 weeks. The short-term primary outcome measure was the mean change in overall ETDQ-7 score from baseline to 6 months. Secondary short-term outcome measures were TM status, tympanogram type, Valsalva maneuver result, and hearing success (defined as ABG ≤ 20 dB from baseline to 6 months). The long-term outcomes were changes from baseline through 30 months postoperatively in the mean overall ETDQ-7 score and middle-ear function tests (TM status, Valsalva maneuver result, mean ABG, and tympanogram type). Improvement in TM status was defined as a change from retracted status (Sade Grade II or III) at baseline to retracted (Sade Grade I or II) or normal status during follow-up, respectively. Improvement in tympanogram type was defined as a change from type B at baseline to types A or C during follow-up, or from type C at baseline to type A during follow-up. Improvement in the Valsalva maneuver result was defined as a change from negative at baseline to positive during follow-up. Statistical analysis was carried out with Student’s *t* test in Microsoft Excel™.

## Results

### Patient demographics

In total, 20 ears of 20 patients with chronic ETD underwent MWA eustachian tuboplasty. However, two ears of two patients were lost to follow-up. Thus, only 18 ears (18 patients) were included in this study. Among the 18 patients (12 women and six men), mean symptom duration was 33.9 ± 8.4 months (range, 24–61 months), and five patients had a previous history of ventilation tube use. The mean patient age was 56.3 ± 4.6 years (range, 42–68 years), and the mean follow-up period was 33.3 ± 2.3 months (range, 30–36 months). The mean preoperative ETDQ-7 score was 29.5 ± 2.8.

No patients experienced severe MWA-related complications during follow-up. Minimal intraoperative bleeding occurred in some patients and was easily cauterized using MWA.

### Surgical outcomes

#### Pre- and postoperative ETDQ-7 scores

The pre- and postoperative ETDQ-7 scores and mean (standard deviation) changes in overall ETDQ-7 score are shown in Table 1. There were statistically and clinically significant improvements in the mean ETDQ-7 score at 6 months postoperatively (change in mean score of 16.7 ± 3.6, *P* < 0.001) and at 30 months postoperatively (change in mean score of 18.9 ± 2.9, *P* < 0.001). Subjective symptoms were not relieved in 16.7% of patients (3/18) at 6 months postoperatively, whereas 88.9% of patients (16/18) exhibited improvement at 30 months postoperatively. In addition, no patients developed symptoms of patulous ET.

**Table 1:** Pre- and post-operative ETDQ-7 score

Patient No.	Side of ear	Pre-op score	Post-op 6 m		Post-op 30 m	
			Score	Change	Score	Change
1	Right	23	12	11	11	12
2	Right	36	11	25	11	25
3	Right	29	12	17	10	19
4	Left	31	12	19	11	20
5	Right	29	10	19	10	19
6	Left	28	10	18	9	19
7	Left	33	10	23	8	25
8	Left	32	11	21	8	24
9	Left	37	28	19	15	22
10	Right	31	12	19	10	21
11	Right	30	11	19	10	20
12	Right	28	9	19	9	19
13	Left	27	23	4	22	5
14	Right	26	11	15	6	17
15	Right	30	14	16	11	19
16	Right	24	10	14	10	14
17	Left	31	15	16	12	19
18	Left	26	10	16	8	18
Mean (SD)		29.5 (2.8)	12.8 (3.2)	16.7 (3.6)	10.6 (2.1)	18.9 (2.9)

#### Audiometric outcomes

**Fig. 3:**
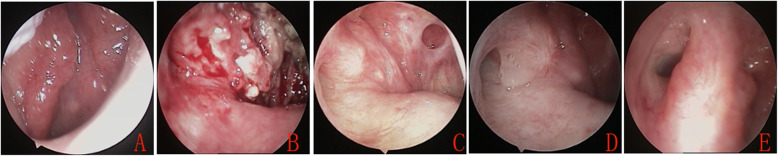
Pre- and postoperative cartilaginous portion of the ET. Preoperative assessment (**a**). Assessments at 2 weeks (**b**), 8 weeks (**c**), 6 months (**d**), and 31 months (**e**) postoperatively

Audiometry outcomes for the 18 patients are shown in Table 2. The mean preoperative ABG was 28.7 ± 2.9 dB (range, 22.5–37.5 dB), whereas it was 19.7 ± 7.3 dB (range, 8.75–33.75 dB) at 6 months postoperatively and 12.8 ± 5.5 dB (range, 6.0–29.5 dB) at 30 months postoperatively (Fig. 3). The hearing success rate (postoperative ABG ≤ 20 dB) was 66.1% (11/18) at 6 months postoperatively and 88.9% (16/18) at 30 months postoperatively. Only one patient (5.6%) exhibited progressive sensorineural hearing loss unrelated to MWA at 8 months postoperatively, which was diagnosed as sudden sensorineural hearing loss; two (11.1%) exhibited stable hearing following surgery.

**Table 2:** Pre-op and post-op mean air-bone gap

Patient No.	Side of ear	Pre-op audiogram findings	Mean ABG (dB HL)		
			Pre-op	Post-op 6 m	Post-op 30 m
1	Right	CHL	28.25	14.75	9.25
2	Right	CHL	31.25	29.75	12.25
3	Right	CHL	29.75	10.25	6.0
4	Left	CHL	22.5	11.25	7.25
5	Right	CHL	26.25	26.5	17.25
6	Left	CHL	22.5	12.75	6.25
7	Left	CHL	26.75	20.25	11.25
8	Left	Mixed HL	28.5	21.25	10.75
9	Left	CHL	28.25	25.5	22.25
10	Right	CHL	29.75	18.55	10.25
11	Right	CHL	35.25	33.25	20.75
12	Right	CHL	37.5	28.5	11.25
13	Left	CHL	26.25	33.25	33.75
14	Right	CHL	25.75	20.5	9.25
15	Right	CHL	31.25	13.5	7.25
16	Right	CHL	32.25	11.25	10.5
17	Left	CHL	24.75	10.25	10.25
18	Left	CHL	29.25	8.75	8.25
Mean (SD)			28.7 (2.9)	19.7 (7.3)	12.8 (5.5)

Mean air–bone gap was calculated from findings at 0.5, 1, 2 and 3/4 kHz. Only changes in mean air–bone gap of 5 dB HL or more were considered significant because of inter-test variability

*Pre-op* Pre-operative, *post-op* Post-operative, *CHL* Conductive hearing loss, *HL* Hearing loss

#### Endoscope observations and middle-ear function

Pre- and postoperative TM status, tympanogram types, and Valsalva maneuver results are summarized in Tables 3 and 4. Of the 18 patients, 13 (72.2%) had TM Sade Grade III and five (27.8%) had TM Sade Grade II before surgery.

**Table 3:** Overview of patient details and outcome of Eustachian tuboplasty

Patient No.	Side of ear	Pre-operative			Post-operative 6 m			Post-operative 30 m		
		Endoscope (Sade Grade)	Tympanometry	Valsalva	Endoscope (Sade Grade)	Tympanometry	Valsalva	Endoscope (Sade Grade)	Tympanometry	Valsalva
1	Right	III	B	(−)	II	C	(+)	II	C	(+)
2	Right	II	B	(−)	Normal	A	(+)	Normal	A	(+)
3	Right	III	B	(−)	II	C	(+)	Normal	A	(+)
4	Left	III	B	(−)	III	B	(−)	III	C	(+)
5	Right	II	B	(−)	Normal	A	(+)	Normal	A	(+)
6	Left	III	C	(−)	II	C	(−)	Normal	A	(+)
7	Left	III	B	(−)	II	C	(+)	Normal	A	(+)
8	Left	II	B	(−)	Normal	A	(+)	Normal	A	(+)
9	Left	III	B	(−)	III	B	(−)	II	C	(−)
10	Right	III	B	(−)	II	C	(+)	Normal	A	(+)
11	Right	II	B	(−)	II	C	(+)	Normal	A	(+)
12	Right	III	B	(−)	II	C	(+)	Normal	A	(+)
13	Left	III	C	(−)	III	B	(−)	III	B	(−)
14	Right	III	B	(−)	II	C	(+)	Normal	A	(+)
15	Right	III	B	(−)	III	B	(−)	I	A	(+)
16	Right	II	B	(−)	Normal	A	(+)	Normal	A	(+)
17	Left	III	C	(−)	I	A	(+)	Normal	A	(+)
18	Left	III	B	(−)	II	C	(+)	Normal	A	(+)

Negative:(−); Positive(+)

**Table 4:** The summary of subjective and objective measure

	Pre-operative	Post-operative 6 m	Post-op 30 m
Mean ETDQ-7 scores	29.5 ± 2.8	12.8 ± 3.2	10.6 ± 2.1
Mean ABG (dB HL)	28.7 ± 2.9	19.7 ± 7.3	12.8 ± 5.5
0–10	0 (0.0%)	3 (16.7%)	12 (66.7%)
11–20	0 (0.0%)	8 (44.4%)	4 (22.2%)
21–30	13 (72.2%)	5 (27.8%)	1 (5.6%)
31–40	5 (27.8%)	2 (11.1%)	1 (5.6%)
TM status			
Sade Grade III	13 (72.2%)	4 (22.2%)	2 (11.1%)
Sade Grade II	5 (27.8%)	9 (50.0%)	2 (11.1%)
Sade Grade I	0	1 (5.6%)	1 (5.6%)
Normal	0	4 (22.2%)	13 (72.2%)
Tympanograms			
Type B	15 (83.3%)	4 (22.2%)	1 (5.6%)
Type C	3 (16.7%)	9 (50.0%)	3 (16.7%)
Type A	0 (0.0%)	5 (27.8%)	14 (77.7%)
Valsalva maneuver			
Negative	18 (100.0%)	5 (27.8%)	2 (11.1%)
Positive	0 (0.0%)	13 (72.2%)	16 (88.9%)
Videoendoscopy of ET			
Complete opening	0	14 (77.7%)	15 (83.3%)
Restrictive opening	0	1 (5.6%)	2 (11.1%)
Failure opening	18 (100.0%)	3 (16.7%)	1 (5.6%)

Pre-tympanogram types were type B in 83.3% of cases and type C in 16.7%.

At 6 months postoperatively, of the 13 patients with an initial TM Sade Grade III, 9 (69.2%) exhibited TM Sade Grade II and an aerated middle ear at 6 months postoperatively, whereas 4 (30.8%) exhibited persistent TM Sade Grade III. Of the five patients with initial TM Sade Grade II, four (80.0%) exhibited normal ears at 6 months postoperatively, whereas one exhibited TM Sade Grade I and an aerated middle ear. The tympanogram was type C in 50.0% of cases, type B in 22.2%, and type A in 27.8%.The Valsalva maneuver results improved from negative at baseline to positive in 13 patients (72.2%), whereas no changes were observed for 5 patients (27.8%). Endoscopic video analysis showed complete opening of the ET valve in 14 patients (77.7%), failure to open in 3 patients (16.7%), and restrictive opening in 1 patient (5.6%). In addition, a small peritubal synechia developed in three patients, which was separated by MWA. Intratubal packing was performed using biodegradable synthetic polyurethane foam for 1 week.

At 30 months postoperatively, 13 patients (72.2%) exhibited normal ears, 2 (11.1%) had TM Sade Grade III, 2 (11.1%) had Sade Grade II, and 1 (5.6%) had an aerated middle ear with TM Sade Grade I and mobility on negative pressure insufflation. The tympanogram was type C in 16.7% of patients, type B in 5.6%, and type A in 77.7%. The Valsalva maneuver results improved from negative at baseline to positive in 16 patients (88.9%), whereas no changes were observed in the remaining two patients (11.1%). Endoscopic video analysis showed complete opening of the ET valve in 15 patients (83.3%) (Fig. 3), restrictive opening in 2 (11.1%), and synechia in 1 (5.6%).

One patient had a type C tympanogram that deteriorated to type B and produced persistently negative Valsalva maneuver results during follow-up, but the TM status remained as Sade Grade III. Another patient had persistent type B tympanogram at 6 months postoperatively and type C at 30 months postoperatively, produced a weak positive Valsalva maneuver, and changed from the initial TM Sade Grade III to Sade Grade II at 30 months postoperatively.

**Fig. 4:**
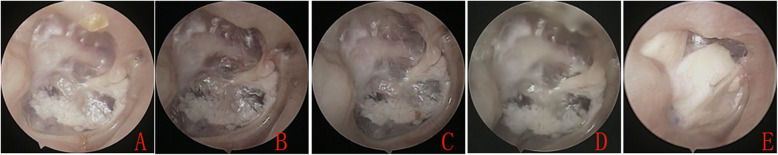
Pre- and postoperative TM status. Preoperative assessment (**a**). Assessments at 2 weeks (**b**), 8 weeks (**c**), 6 months (**d**), and 31 months (**e**) postoperatively

Interestingly, all 13 patients with normal TM showed increased myringosclerosis extension of the pars tensa at 30 months postoperatively, and 38.5% of patients (5/13) exhibited complete sclerosis of the pars tensa (Fig. 4).

## Discussion

There is accumulating evidence that hypertrophy involving the cartilaginous portion of the ET is the main cause of chronic ETD. Therefore, some researchers have proposed that endoscopic intraluminal surgery could have potential for improving ET dilation and middle ear ventilation. Poe et al. [2] and Metson et al. [5] performed laser and microdebrider eustachian tuboplasty, respectively, to remove hypertrophied mucosal and submucosal tissue from the posterior wall of the cartilaginous portion and reduce the volume to dilate the tubal lumen. In recent years, balloon eustachian tuboplasty has become popular and has produced promising results in studies worldwide [6–9, 19]. Notably, this method has had limited application in some countries and areas because of its high cost.

Here, we performed MWA eustachian tuboplasty in patients with chronic ETD and TM retraction. MWA was initially developed in the early 1980s to achieve hemostasis along the plane of transection during hepatic resection and has been confirmed to be effective for the treatment of acute hemorrhage [11]. MWA allows the microwave antenna in the target tissue to rapidly achieve an average local temperature of 65 °C–100 °C [3], which allows the temperature of the surrounding tissue to become considerably higher, thus covering a larger tissue volume. This results in a larger ablation zone within a shorter time [11–13]. In recent years, MWA has been widely used to treat hypertrophic rhinitis [20] and epistaxis [14, 17] as well as to remove benign nasal and pharyngeal lesions [15, 16]. In the present study, the overall ETDQ-7 scores and objective outcomes improved over time, i.e., from 6 to 30 months, following MWA tuboplasty. However, while the improvement of Valsalva maneuver was relatively stable at 6 and 30 months, abnormal tympanograms took 30 months to show maximum improvement. This is not surprising because the intraluminal mucosal edema was gradually relieved, and resulted in restricted aeration of the middle ear and ET in some patients at 6 months postoperatively, while aeration improved completely and the TM recovered its normal activity over time. It is interesting that the ET function improved over time from 6 to 30 months, suggesting that surgeons performing ET procedures may need to advise their patients that it can take more than a year to achieve the final improvement.

Metson et al. [5] and Caffier et al. [3] reported that abnormal tympanography results improved in 65% of cases using microdebrider tuboplasty and 66% of cases using laser tuboplasty, respectively, at 1 year postoperatively. McMurran et al. [8] found significant improvements using the balloon dilation technique in postoperative ETDQ-7 score, abnormal tympanography results, and TM status in 64, 64, and 50% of patients, respectively. Some groups reported that 66–72% of patients achieved normal middle ear aeration following laser tuboplasty at 1 year [2–4]. Although balloon tuboplasty has some advantages, such as a short operation time, good tolerability, lack of mucosal injury of the anterior tubal wall and significant complications, as well as improvements in long-term subjective and objective measures [6–9], Wang et al. [21] reported that Valsalva maneuver results were similar between the balloon and laser procedures.

The results of the present study were better than those reported previously [3, 5, 8]. This could be related to the duration of follow-up, because the mean follow-up was 6–12 months in most previous studies of laser [3], microdebrider [5], or balloon tuboplasty [8] for ETD, whereas it was 30 months in the present study. Silvola et al. [6] also reported 30 months of follow-up after the balloon dilation technique, and 80% of their patients could perform a Valsalva maneuver postoperatively, while subjective symptoms were relieved in 90% of patients and all patients exhibited improved tympanometry results. Using laser eustachian tuboplasty, Kujawski et al. [22] reported tympanogram type A and normal TM status in 58.3% of patients (63/108) at 1 year postoperatively, and in 60.9% (56/92) at 3 years postoperatively. Yañez et al. [23] used cross-hatching tuboplasty and reported that tympanometry improved at 1, 2, and 5 years postoperatively in 88.9% (176/198), 92.9% (184/198), and 93.4% (185/198) of patients, respectively.

The mechanism of MWA tuboplasty is similar to microdebrider and laser techniques, which surgically remove or ablate the mucosa, submucosa, and soft tissue in the posterior cushion of the ET orifice and posterior wall of the tubal lumen. It can substantially reduce the mucosal and submucosal thickness of the posterior cushion and wall, and cause postoperative mucosa fibrosis, thereby facilitating ET dilation and improving ET function [2, 3, 5, 24]. The MWA technique is theoretically superior to microdebrider and laser techniques. The microdebrider blade (diameter, 2.9–4 mm) cannot easily reach the valve area [5], while the microwave antenna tip can easily reach the valve area. In addition, the microdebrider technique causes intraoperative bleeding and the laser technique produces black crust, both of which affect the surgical field and lead to incomplete lesion removal. Conversely, MWA allows both lesion removal and hemostasis using the same instrument. It forms a gray-white ablated zone to ensure a clear surgical field for valve identification. In addition, tubal lumen dilation was greater with further development of submucosal fibrosis. A few studies found that the improvement in ET function following tuboplasty is not immediate but emerges after a period of 6–8 weeks [24]. Nevertheless, the high rate of effectiveness in the present study may have partially resulted from the strict inclusion criteria. Only patients with a retracted TM were selected in this study, whereas patients with protracted otitis media and effusion, with or without sinonasal disease, were included in previous studies [2–6]. Notably, the degrees of ET stenosis and mucosal hyperplasia in patients with a retracted TM were greater than those in patients with otitis media and effusion, resulting in a greater degree of improvement.

MWA tuboplasty differs from ET balloon tuboplasty, and is used to ablate the hyperplastic mucosa and submucosa of the ET orifice. However, a balloon is inserted into the ET and inflated to dilate the cartilaginous ET in balloon tuboplasty, in which the mechanical force of balloon dilation produces submucosal microhemorrhages, thereby resulting in postoperative fibrosis that expands the luminal diameter as tissue healing progresses [19, 25, 26]. The balloon is available in diameters of 3.28–7 mm and lengths of 2 and 20 mm in balloon dilatation systems [7, 9], while the microwave antenna tip is available in a diameter of 1 mm and length of 2 mm, with which it is almost impossible to perform ET dilatation.

Abnormal TM status improved in 77.8% of patients (14/18) at 6 months postoperatively and in 94.4% of patients (17/18) at 30 months postoperatively. Surprisingly, the extent of sclerotic plaque increased over time, whereas the extents of atrophy and tensa retraction decreased. These findings were not reported in previous tuboplasty studies but have been observed in studies of ventilation tube or myringotomy usage [27]. In the present study, sclerotic plaque extension increased in 13 patients (72.2%) at 30 months postoperatively. Of these 13 patients, five (38.5%) exhibited complete sclerosis of the pars tensa. The mechanisms and clinical importance of increased myringosclerotic plaque following MWA tuboplasty are unclear. Some groups suggested that an inherent TM repair mechanism was initiated to maintain consistent dynamic eardrum properties through increased myringosclerosis plaque following myringotomy and VT, but it does not influence the short- or long-term tympanometric profile or ET function [27]. Nevertheless, the results may be improved by eustachian tuboplasty in most patients, as in the present and previous studies [2–9]. In the present study, the increase in extent of myringosclerosis plaque was thought not to have been due to the MWA technique, but rather to be a result of natural continuation of the disease process with ET ventilation following tuboplasty. However, further studies are required to determine whether energy can be transferred to the TM to cause myringosclerosis.

In this study, one patient produced persistently negative Valsalva maneuver results and did not experience any subjective relief of their symptoms during the follow-up period. Another patient had only weak positive Valsalva maneuver, and the TM changed from an initial TM Sade Grade III to Sade Grade II at 30 months postoperatively. The intraluminal overlying mucosa in the valve area might have been overly ablated, thus inducing intraluminal synechia and aggravating ET function. Other unidentified factors might have contributed to the poor outcome in this patient, such as cartilaginous abnormalities or alteration of the tensor veli palatini or levator veli palatini muscle, etc. [2, 19]. Careful ablation should be performed with respect to hypertrophied mucosal and submucosal tissue in the posterior intraluminal wall and valve area, while avoiding mucosal injury to the anterior tubal wall.

The safety of MWA in the treatment of nasal cavity and pharyngeal lesions has been evaluated. Previous studies suggested that MWA was safe and reliable for the treatment of nasal cavity and pharyngeal lesions, with no MWA-related complications or injury to surrounding structures [13–16, 28]. There were no surgical complications in this study. The thermal lesion was approximately 2 mm in length and approximately 1 mm in width, whereas the penetration depth was 0.5–1 mm for each ablation in this study [15, 16]. The thermal lesion size could be completely controlled by a footplate-operated switch, which may have reduced the risk of complications. In addition, the medial cartilaginous laminae were not penetrated with the MWA. Thus, the cartilage provided a safety margin for avoiding direct carotid injury. Throughout the procedure, all tissue removal remained superficial to this cartilage. Nevertheless, it should be noted that placing the probe into the lumen and heating it could result in scarring and worsening function. Unfortunately, there have been no cadaver studies of the safety of MWA in terms of the neurovascular structures of the head and neck. However, it is worth noting that 17% of our patients (3/18) had synechiae requiring revision surgery. Although this represents a minor risk, dynamic follow-up by endoscopy should be encouraged.

Our study was designed as a pilot investigation to establish the efficacy of this new technique. Larger studies with additional comparisons are needed to draw more definitive conclusions. Future studies should include prospective case-control designs. In addition, this study was limited by the lack of a validated grading scale of ET opening and collection of inter-rater reliability data.

## Conclusions

MWA eustachian tuboplasty is a feasible alternative to conventional tuboplasty, which allows continued improvement of the subjective and objective outcomes in patients with ETD for up to 30 months following tuboplasty. In addition, this study showed that the extent of sclerotic plaque increased over time, whereas the extents of atrophy and tensa retraction decreased following tuboplasty in most patients.

## Data Availability

The datasets supporting our conclusions are included within the article.

## Abbreviations

ABG: Air-bone gap
ET: Eustachian tube
ETD: Eustachian tube dysfunction
ETDQ-7: ETD-7 questionnaire
MWA: Microwave ablation
TM: Tympanic membrane
